# Two-Year Follow-Up of Clinical Efficacy of Femtosecond Laser, Modified Capsular Tension Ring, and Iris Hook-Assisted Surgical Treatment of Lens Subluxation in Patients with Elevated Intraocular Pressure

**DOI:** 10.1155/2022/4810103

**Published:** 2022-05-09

**Authors:** Chao Wang, Yuhua Rui, Yi Zhou, Tu Hu, Xiaobo Xia, Jian Jiang

**Affiliations:** ^1^Eye Center of Xiangya Hospital, Central South University, Changsha, Hunan, China; ^2^Hunan Key Laboratory of Ophthalmology, Changsha, Hunan, China; ^3^National Clinical Research Center for Geriatric Diseases, Xiangya Hospital, Central South University, Changsha, Hunan, China

## Abstract

**Purpose:**

To evaluate the outcomes of femtosecond laser, modified capsular tension ring, and iris hook-assisted surgical treatment of lens subluxation in patients with elevated intraocular pressure (IOP).

**Methods:**

Fifteen patients with lens subluxation and elevated IOP were enrolled in this study. All patients underwent femtosecond-laser-assisted cataract surgery/phacoemulsification/intraocular lens implantation/modified capsular tension ring (MCTR) implantation with iris hook assistance. Uncorrected visual acuity (UCVA), best corrected visual acuity (BCVA), IOP, number of glaucoma medication complications, endothelial cell density (ECD), and tilt of the lens were recorded before and after surgery. All patients were observed for 24 months postoperatively.

**Results:**

UCVA and BCVA increased significantly at 1 month, 6 months, 12 months, and 24 months, compared with preoperative UCVA and BCVA (*P* < 0.001). IOP significantly decreased at 1 month, 3 months, 6 months, 12 months, and 24 months, compared with preoperative IOP (*P* < 0.001). 3 patients received glaucoma medications to control IOP after surgery. All medications were discontinued at 3 months postoperatively. Conjunctival redness or hemorrhage was observed in 11 patients (73.3%); transient corneal edema was observed in 3 patients (20.0%); and posterior capsule opacification occurred in 1 patient (6.67%). The ECD and tilt of the lens are within an acceptable range.

**Conclusion:**

The combined use of a femtosecond laser, MCTR, and iris hooks is an effective and safe method for treating patients with lens subluxation and elevated IOP.

## 1. Introduction

Lens subluxation is a type of ectopia lentis that presents as a partial dislocation of the lens. It is a common eye disease that could be congenital or acquired. Congenital diseases such as Marfan syndrome are considered to cause altered fibrillin microfibrils which will lead to abnormalities of zonular fibers and lens capsule [1]. Congenital aniridia is a rare bilateral pan ocular disorder and is associated with lens subluxation [2]. The acquired lens subluxation can be traumatic and spontaneous. Blunt trauma is the most common cause. Spontaneous lens subluxation secondary to other ocular diseases such as glaucoma, high myopia, retinal detachment, and pseudoexfoliation syndrome. Postcataract surgery lens subluxation is also a common issue for clinical management. Occult lens subluxation could also be secondary to laser peripheral iridotomy [3]. The major changes in lens subluxation of different causes are typical: partial tearing or loosening of zonular ligaments and dislocation of the lens. Lens subluxation can cause impaired vision and even lead to dangerous complications if left unt\]reated. The dislocated lens becomes spherical because of its own elasticity, thereby increasing its anteroposterior diameter which will lead to pupillary block, shallow anterior chamber, and anterior chamber angle closure. Acquired lens subluxation can also be accompanied by vitreous prolapse. Therefore, elevated IOP is common and critical in patients with lens subluxation [4, 5]. Clinical treatments for lens subluxation are complex. Patients who received inappropriate or delayed treatments may exhibit uncontrollable elevated IOP and require additional surgeries. With the advent of extracapsular cataract extraction and intraocular lenses (IOLs), intracapsular cataract extraction was replaced during lens subluxation treatment [6]. Practical surgical considerations should include the removal of the lens and its capsule as well as the preservation of IOL stability. The challenges of lens subluxation treatment mainly involve difficulty with continuous curvilinear capsulorhexis (CCC), as well as poor lens stability, vitreous prolapse, and serious complications [7].

Recent advances in surgical techniques allow for combined application of femtosecond-laser-assisted cataract surgery (FLACS)/phacoemulsification (PE)/IOL implantation/modified capsular tension ring (MCTR) implantation surgery with iris hook assistance that greatly improved the surgical safety and outcomes of lens subluxation. The application of femtosecond laser technology leads to less damage, facilitates CCC, improves accuracy, and aids in achieving good visual quality [8, 9]. Iris hooks greatly improve the stability of the capsular bag, which aids in PE and IOL implantation [10]. MCTR implantation improves the stability of the capsular bag [11]. By combining these three techniques, the operation is safe and IOP can be controlled in an optimal manner. In this retrospective study, we evaluated the outcomes of the combined application of these three techniques in treating patients with lens subluxation and elevated IOP and provided guidance for future clinical management.

## 2. Materials and Methods

This comparative, retrospective, cohort study included patients with lens subluxation and elevated IOP from November 2018 to May 2021. Informed consent was obtained from all patients recruited prior to the study. Patient-specific surgical procedures were determined by the treating physicians and were performed according to the standard-of-care of our hospital. Deidentified medical records were screened and extracted between 12^th^ May 2021 and 16^th^ May 2021. This study was approved by the clinical medical ethics committee of Xiangya Hospital, Central South University, and adhered to the tenets of the Declaration of Helsinki.

All 15 patients recruited had elevated IOP; 10 of them had traumatic lens subluxation and 5 of them had idiopathic lens subluxation. Detailed demographic information is provided in Table 1. All patients underwent FLACS/PE/IOL implantation/MCTR implantation surgery with iris hook assistance. 6 patients exhibited vitreous prolapse in the anterior chamber, which required anterior vitrectomy. An astigmatic keratotomy was performed in 1 patient to correct astigmatism. All surgeries were performed by a single skilled surgeon (JJ). Patient characteristics were recorded as follows: UCVA BCVA, IOP before the surgery and 1 day, 1 month, 3 months, 6 months, 12 months, and 24 months after surgery; use of antiglaucoma treatment before and after surgery; postoperative complications, ECD, and tilt of the lens; type of capsular tension device used and type of IOL implanted.

### 2.1. Surgical Technique

Anterior angle, degree of lens subluxation, and pupil status were evaluated carefully before the surgery. Topical levofloxacin (Cravit; Santen, Japan) was applied to the eye scheduled for surgery, four times a day for 3 days before surgery. Tropicamide phenylephrine (Cravit; Santen, Japan) was used to dilate pupils 30 minutes before surgery. The patient's head position was fixed and ocular surface was anaesthetised with oxybuprocaine hydrochloride eye drops (Cravit; Santen, Japan). FLACS was performed using the Victus platform (Bausch + Lomb, Rochester, NY, USA) with conventional femtosecond laser settings. Femtosecond laser-assisted astigmatic keratotomy was performed in 1 patient to correct astigmatism. For FLACS, a 5.0 mm-diameter capsulotomy with pupil centration was performed, following a concentric cylinder and a chop (sextants cut) pattern for lens fragmentation. A 2.8 mm transparent corneal incision and a corneal side incision were created with a disposable keratome. The anterior capsule was then removed, and Viscoelastic (Bausch + Lomb, Rochester, NY, USA) was injected into the anterior chamber. A 15° knife was used to make five limbal incisions at intervals of approximately 75°. Five iris hooks were attached to the edge of the capsule through the incisions, which allowed fixation and centering of the lens. Patients who exhibited intraoperative vitreous prolapse in the anterior chamber underwent anterior vitrectomy.

**Figure 1:**
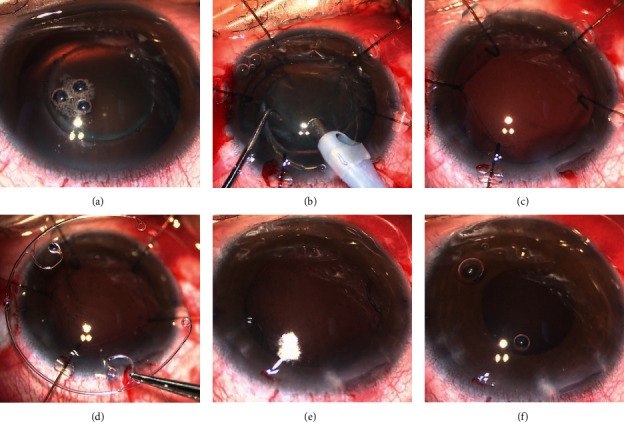
(a) Capsulotomy by femtosecond laser centred on the capsular bag. (b, c) Five iris hooks were attached to the edge of the capsule through the incisions to ensure fixation and centering during the PE procedure. (d) The MCTR was implanted in the capsular bag. ((e), (f)) Iris hooks were removed, and the anterior chamber viscoelastic agent was aspirated. Hydration was performed to seal the clear corneal incisions.

For the PE procedure, we used Stellaris PC (vitreous cutter; Bausch + Lomb, Rochester, NY, USA) and Centurion® Vision System (Alcon Laboratories, Inc., Fort Worth, TX, USA) for all patients and kept system settings consistent. Sufficient hydrodissection and hydrodelineation were carefully performed to reduce the pressure on zonular ligaments during the PE process. Without rotation of the nucleus, the cortex was separated along femtosecond cleavage planes and aspirated with the PE probe. A chopper was applied to resist influences caused by negative pressure on the capsular bag. Low flow, low perfusion pressure, low negative pressure, and low ultrasonic energy were kept during PE (Figure 1).

**Figure 2:**
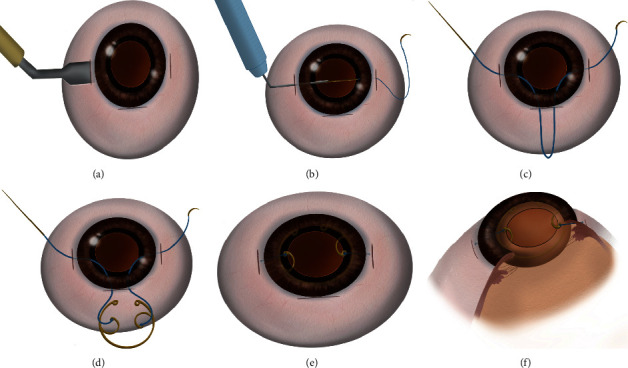
(a) Nasal and temporal scleral tunnels were made by a crescent knife at 2.0 mm behind the limbus. (b) A 9–0 polypropylene suture was threaded through scleral tunnels. (c) The suture was pulled out from the main incision carefully. (d) The suture was cut in half and attached to the fixation hooks of MCTR. (e, f) MCTR was implanted in the capsular bag, and the suture was knotted in the scleral tunnel.

Polymethylmethacrylate MCTR (Morcher GmbH, Stuttgart, Germany) was implanted in the capsular bag, fixation hooks were attached to a 9–0 polypropylene suture (Ethicon, Somerville, NJ, USA) and knotted in the prescleral tunnel 2.0 mm behind the limbus (Figure 2). The IOL (Tecnis ZCB00 or Rayner920) was implanted in the capsular bag using a syringe, iris hooks were then removed, and the anterior chamber viscoelastic agent was aspirated. Hydration was then performed to seal the clear corneal incisions.

### 2.2. Postoperative Protocol

The following topical medication regimen was prescribed: levofloxacin eye drops and prednisolone acetate (Allergan, Irvine, CA, USA) eye drops, 4 times/day for 2 weeks; pranoprofen eye drops (Senju Pharmaceutical Ltd., Osaka, Japan), 4 times/day for 1 month. 2 patients were prescribed with Timolol (Wujing, Wuhan, China) for IOP control, 2 times/day for 3 months. Postoperative examinations and data collection were performed as mentioned in previous section.

### 2.3. Statistical Analysis

All data were analysed using PASW 18.0 (SPSS Inc., Chicago, IL, USA). Results are reported as the mean ± standard deviation. Paired *t*-test was used for repeat testing of UCVA, BCVA, IOP, and ECD in each patient. Differences were considered to be statistically significant when *P* < 0.05.

## 3. Results

15 patients (15 eyes) with lens subluxation were recruited in this study. 10 patients exhibited traumatic lens subluxation and 5 patients exhibited idiopathic lens subluxation. All patients had elevated IOP. Detailed patient information is shown in Table 1. There were 10 male and 5 female, with an average age of 52.40 years (median: 53 years; range: 38–65 years). All patients were followed up for 24 months with one exception case that was lost to follow-up at 6 months. All patients underwent FLACS/PE/IOL implantation/MCTR implantation surgery. 6 of them underwent additional anterior vitrectomy due to vitreous prolapse in the anterior chamber (Table 1).

**Figure 3:**
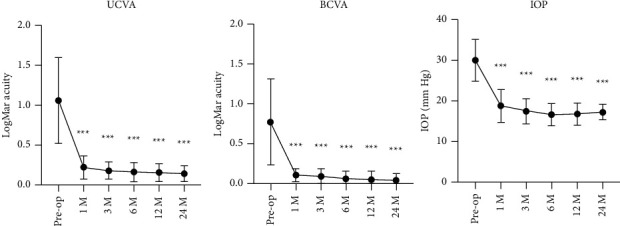
UCVA and BCVA significantly increased at 1 month, 3 months, 6 months, 12 months, and 24 months, compared with preoperative UCVA (*n* = 15, ^*∗∗∗*^*P* < 0.001, ^*∗∗*^*P* < 0.005). IOP significantly decreased at 1 month, 3 months, 6 months, 12 months, and 24 months, compared with preoperative IOP (*n* = 15, ^*∗∗∗*^*P* < 0.001).

UCVA increased significantly at 1 month (0.22 ± 0.14, *P* < 0.001), 3 months (0.18 ± 0.11, *P* < 0.001), 6 months (0.16 ± 0.12, *P* < 0.001), 12 months (0.16 ± 0.11, *P* < 0.001), and 24 months (0.14 ± 0.10, *P* < 0.001) postoperatively, compared with their preoperative UCVA (1.06 ± 0.54). BCVA increased significantly at 1 month (0.10 ± 0.08, *P* < 0.001), 3 months (0.09 ± 0.09, *P* < 0.001), 6 months (0.06 ± 0.10, *P* < 0.001), 12 months (0.05 ± 0.10, *P* < 0.001), and 24 months (0.04 ± 0.09, *P* < 0.001) postoperatively, compared with preoperative BCVA (0.77 ± 0.54) (Table 2 and Figure 3).

IOP significantly decreased at 1 month (18.87 ± 4.03 mm·Hg, *P* < 0.001), 3 months (17.53 ± 3.09 mm·Hg, *P* < 0.001), 6 months (16.71 ± 2.70 mm·Hg, *P* < 0.001), 12 months (16.87 ± 2.75 mm·Hg, *P* < 0.001), and 24 months (17.33 ± 1.84 mm·Hg, *P* < 0.001) postoperatively, compared with preoperative IOP (30.13 ± 5.15 mm·Hg). 3 patients required postoperative glaucoma medications to control IOP and all medications were discontinued at 3 months after surgery. All patients required glaucoma medications to maintain IOP in the normal range before surgery. Combined application of timolol and brimonidine tartrate eye drops (Allergan) was prescribed for treating patients with IOP >30 mm·Hg, while timolol was used alone for IOP within 21–30 mm·Hg. Three patients required postoperative use of timolol to control IOP. At 3 months postoperatively, all patients exhibited optimal IOP control without any glaucoma medications (Table 3 and Figure 3).

Conjunctival redness or hemorrhage was observed in 11 patients (73.3%). Transient corneal edema was observed in 3 patients (20.0%) and posterior capsule opacification occurred in 1 patient (6.67%). Descemet's membrane detachment, lens material in vitreous, posterior capsule rupture, and iris damage were not observed in all 15 patients (Table 4).

Decreased ECD was observed in all 15 patients at 24 months (2182.67 ± 138.84) after surgery compared with preoperative ECD (2294.40 ± 142.84, *P* < 0.001) but the differences were within the normal range. Visual quality was assessed using OPD Scan III (Nidek Inc., Tokyo, Japan) at 24 months after surgery. The tilt of the lens (<1) is within an acceptable range, and there is no effect on vision acuity (Table 5 and Table 2).

**Figure 4:**
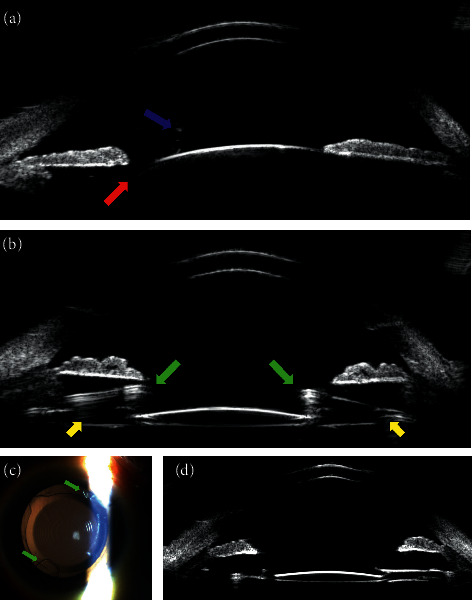
(a) Before surgery, the subluxated lens (red arrow) and vitreous entering the anterior chamber (blue arrow) were detected by ultrasound biomicroscopy. (b) After surgery, the IOL and MCTR were located in the correct positions. Fixation hooks of MCTR (green arrows), haptics of IOL, and MCTR were also observed (yellow arrows). (c) After surgery, extended depth of focus IOL and fixation hooks of MCTR (green arrows) were observed in a slit-lamp examination. (d) Ultrasound biomicroscopy showed the extended depth of focus IOL in the correct position.

Ultrasound biomicroscopy (UBM) prior to the surgery revealed subluxated lens and vitreous entering the anterior chamber whereas after the surgery, the IOL and MCTR were in correct positions (Figure 4).

## 4. Discussion

A subluxated lens deviates from the visual axis, such that affected patients may exhibit astigmatism or monocular diplopia. During mydriatic examination, the equatorial portion of the lens and iris tremor is visible in the pupil area. In patients with lens subluxation, dislocation of the lens will lead to iris pushing forward, and frequently cause a shallow anterior chamber and elevated IOP. Vitreous prolapse and trabecular meshwork injury caused by trauma can also contribute to IOP elevation. Surgical treatment of lens subluxation is often considered difficult and has been considered as a contraindication for PE surgery. In the past, lens subluxation was treated by intracapsular cataract extraction, extracapsular cataract extraction with IOL suspension, or anterior chamber IOL implantation. These surgical methods, though proved to be effective, can be complicated in procedures. Along with prolonged operative times and large incisions, these methods may cause many complications, such as corneal endothelial decompensation and secondary glaucoma [6, 12, 13]. Notably, PE disturbs intraocular tissue, thereby enhancing the extent of lens dislocation and lens ligament damage. Due to a lack of adequate support, IOLs implanted are often eccentric, dislocated, or trapped by the pupil. These factors can also potentially reduce the safety of the surgical procedures.

The development of microsurgical techniques and equipment has improved our understanding of lens subluxation treatment. The combined application of FLACS, MCTR, and iris hook methods has been shown to greatly improve the safety and postoperative outcomes of lens subluxation treatment [14, 15]. The use of a preserved capsular bag can minimise disturbance to the vitreous body, while creating good conditions for IOL implantation. Furthermore, this technique can reduce the occurrence of complications such as retinal detachment and secondary glaucoma [16].

Regarding CCC, femtosecond laser-assisted in situ anterior lens capsule incision has several advantages including more accurate capsulotomy with a well-centered and an approximate ring shape. The process is also repeatable and predictable [17–19]. For patients with lens subluxation, femtosecond laser-assisted in situ anterior lens capsulotomy and laser cleavage can minimise surgical damage to the lens ligament. With this method, the lens capsular bag can be successfully protected in 90% of the eyes [8, 9, 20]. However, femtosecond laser treatment is not recommended for eyes with a large dislocation range and a capsulotomy area blocked by the iris. Our results indicate that the anterior vitreous has less influence on CCC, as shown in a prior study [21]. Notably, the IOP could be elevated due to the docking procedure during the femtosecond laser process and presumably returns to the normal range within a short period of time [22].

Maintenance of capsular bag stability is an important consideration throughout the surgical process. Stability of the capsular bag and good surgical field of view are critical for reducing surgical complications [23–25]. Iris hooks can restore the subluxated lens to its normal physiological position, fully dilate the capsular bag, prevent further damage to the lens ligament and capsular bag, reduce disturbance to the vitreous, avoid vitreous prolapse, and reduce intraoperative and postoperative complications. Iris hooks also provide a good surgical viewing field and stable operative space for PE, MCTR implantation, and IOL implantation. Moreover, incisions from iris hooks have minimal effects on corneal astigmatism.

Sufficient hydrodissection and hydrodelineation during PE are essential procedures for reducing the pressure on zonular ligaments. For maintaining capsular stability, nucleus rotation is not recommended. Appropriate managements include using low negative pressure and low aspiration power and lowering the height of the bottle appropriately.

Implementation of a capsular tension ring (CTR) is an effective solution to the problems involved in lens subluxation mentioned above. CTRs compensate for weak ligaments in multiple manners. For weak or broken focal ligaments, CTRs can redistribute mechanical force to the ligaments, thereby stretching the equatorial portion of the capsular bag outward. CTRs can also resist postoperative contraction of the capsular bag, reduce the incidence of posterior capsular opacification, and enhance the stability of IOLs [26–28]. Regarding the timing of CTR implantation, CTR implantation earlier than PE can significantly enhance the capsular torque, such that displacement can reach 4.0 mm. We chose to implant MCTR after PE to minimise ligament stress and damage. The intrascleral fixation of MCTR simplifies the surgical procedure and avoids the fraction between the knot and conjunctiva. This technique was also applied in fixation of IOL and achieved ideal outcomes [29]. Compared with traditional CTR implantation, MCTR implantation can avoid IOL displacement caused by progressive abnormal lens ligament and resist CTR displacement resulting from capsular fibrosis and shrinkage.

Successful CCC and good capsular bag fixation provide a good foundation for IOL implantation. Selection of the appropriate IOL is of considerable importance for avoiding long-term complications. Notable long-term postoperative complications include off-centre movement, tilting, or displacement of the IOL; common causes of these complications are asymmetric contraction of the capsular bag, as well as progressive tearing or loosening of the zonular ligaments. Based on the above considerations, mono focal aspherical IOLs were used in 7 eyes (87.5%) in this study, while the extended depth of focus IOL was used in 1 eye (12.5%). During follow-up, no obvious capsular bag contraction or IOL rotation were observed; postoperative vision recovery was stable. In recent years, many studies have confirmed that FLACS combined with CTR implantation can be used to implant many high-tech IOLs, such as toric IOLs and even trifocal IOLs [26, 28]. We chose the extended depth of focus IOL for 1 patient, and the satisfied outcome supports our use of high-tech IOLs to help patients achieve better visual outcomes in the future.

## 5. Conclusions

Our findings indicate that combined FLACS/PE/IOL implantation/MCTR implantation surgery with iris hook assistance provides simpler procedures, a more stable process, and better visual quality. This surgical technique is an effective and safe method for treating patients with lens subluxation and elevated IOP to improve UCVA and achieve optimal IOP control.

## Figures and Tables

**Table 1:** Demographic and clinical characteristics of eyes in this study.

Patient	Sex	Follow- up (M)	Age (Y)	Eye	Etiology	Clock hours	Vitreous prolapse	IOL	Surgical procedure
1	M	24	50–54	OS	Trauma	5	−	ZCB00	FLACS/PE/IOL/MCTR
2	M	24	35–40	OD	Trauma	6	+	Rayner920	FLACS/PE/IOL/MCTR/Vitrectomy
3	F	24	40–44	OD	Trauma	5	−	ZCB00	FLACS/PE/IOL/MCTR
4	M	24	55–60	OD	Idiopathic	5	−	Rayner920	FLACS/PE/IOL/MCTR
5	F	24	55–60	OS	Idiopathic	6	−	Rayner920	FLACS/PE/IOL/MCTR
6	M	24	60–64	OD	Trauma	6	+	ZCB00	FLACS/PE/IOL/MCTR/Vitrectomy
7	M	24	65–69	OD	Trauma	5	−	ZCB00	FLACS/PE/IOL/MCTR
8	M	24	50–54	OD	Idiopathic	5	+	ZXR00	AK/FLACS/PE/IOL/MCTR/Vitrectomy
9	F	24	55–60	OD	Trauma	5	+	Rayner920	FLACS/PE/IOL/MCTR/Vitrectomy
10	M	24	45–50	OS	Trauma	5	−	Rayner920	FLACS/PE/IOL/MCTR
11	M	24	45–50	OS	Trauma	5	−	ZCB00	FLACS/PE/IOL/MCTR
12	M	24	40–44	OD	Trauma	6	+	ZCB00	FLACS/PE/IOL/MCTR/Vitrectomy
13	M	24	45–50	OS	Trauma	6	+	Rayner920	FLACS/PE/IOL/MCTR/Vitrectomy
14	F	24	55–60	OD	Idiopathic	5	−	ZCB00	FLACS/PE/IOL/MCTR
15	F	24	55–60	OD	Idiopathic	5	−	ZCB00	FLACS/PE/IOL/MCTR

**Table 2:** Postoperative UCVA and BCVA.

Patient	UCVA (logMAR)						BCVA (logMAR)					
	Preop	1 M	3 M	6 M	12 M	24 M	Preop	1 M	3 M	6 M	12 M	24 M
1	0.70	0.05	0.10	—	0.10	0.10	0.40	0.00	0.00	—	0.00	0.00
2	1.70	0.40	0.30	0.22	0.22	0.22	1.22	0.05	0.05	0.05	0.05	0.05
3	1.00	0.15	0.15	0.15	0.15	0.15	0.70	0.10	0.10	0.10	0.10	0.10
4	0.52	0.15	0.05	0.00	0.00	0.00	0.52	0.05	0.00	−0.08	−0.08	0.00
5	1.40	0.22	0.30	0.22	0.30	0.22	1.10	0.22	0.22	0.15	0.15	0.15
6	1.22	0.52	0.30	0.40	0.40	0.30	1.22	0.22	0.22	0.22	0.22	0.10
7	0.70	0.10	0.10	0.05	0.05	0.05	0.70	0.05	0.00	0.00	0.00	−0.08
8	2.30	0.30	0.22	0.22	0.22	0.15	2.30	0.10	0.00	−0.08	−0.08	-0.08
9	1.70	0.30	0.22	0.30	0.22	0.30	0.70	0.22	0.22	0.22	0.22	0.22
10	0.70	0.15	0.15	0.10	0.15	0.10	0.30	0.05	0.05	0.05	0.05	0.05

**Table 3:** Postoperative IOP and treatment results.

Patient	IOP (mmHg)						Postop antiglaucoma treatment
	Preop	1 M	3 M	6 M	12 M	24 M	
1	25	21	18	—	17	17	No
2	28	15	19	14	14	16	No
3	30	22	20	21	19	19	No
4	29	17	19	20	20	20	No
5	35	25	20	18	16	15	Yes
6	32	23	21	15	15	14	Yes
7	26	22	16	18	19	18	No
8	25	18	16	16	16	16	No
9	33	11	10	12	12	16	No
10	31	20	20	17	20	20	No
11	27	19	21	18	19	18	No
12	24	13	14	15	13	16	No
13	42	23	18	17	18	19	Yes
14	38	19	17	20	21	19	No
15	27	15	14	13	15	17	No

**Table 4:** Postoperative complications.

Patient	Conjunctival redness or hemorrhage	Descemet's membrane detachment	Lens material in vitreous	Posterior capsule rupture	Transient corneal edema	Iris damage	Posterior capsule opacification
1	+	−	−	−	−	−	−
2	+	−	−	−	−	−	−
3	+	−	−	−	−	−	−
4	−	−	−	−	−	−	−
5	−	−	−	−	−	−	−
6	+	−	−	−	+	−	−
7	+	−	−	−	−	−	−
8	−	−	−	−	−	−	−
9	+	−	−	−	+	−	−
10	+	−	−	−	−	−	−
11	+	−	−	−	−	−	−
12	+	−	−	−	+	−	−
13	+	−	−	−	−	−	−
14	+	−	−	−	−	−	−
15	−	−	−	−	−	−	+

**Table 5:** Postoperative ECD and tilt of the lens.

Patient	ECD (Cells/mm^2^)		IOL tilt
	Preop	Postop 24 m	Postop 24 m
1	2212	2101	0.774@89°
2	2398	2184	0.912@132°
3	2247	2082	1.003@233°
4	2301	2289	0.691@279°
5	2564	2437	0.847@23°
6	2218	2045	0.935@16°
7	2435	2341	0.787@63°
8	2497	2318	0.669@81°
9	2121	2058	0.894@168°
10	2230	2155	0.658@313°
11	2291	2201	0.992@245°
12	2206	2085	0.792@92°
13	2019	1916	0.805@58°
14	2387	2310	0.975@113°
15	2290	2218	0.881@328°

## Data Availability

The data supporting the findings of this study are available within the article.
